# Inter-basin sources for two-year predictability of the multi-year La Niña event in 2010–2012

**DOI:** 10.1038/s41598-017-01479-9

**Published:** 2017-05-23

**Authors:** Jing-Jia Luo, Guoqiang Liu, Harry Hendon, Oscar Alves, Toshio Yamagata

**Affiliations:** 1 Bureau of Meteorology, Melbourne, Australia; 2 Application Lab, JAMSTEC, Yokohama, Japan

## Abstract

Multi-year La Niña events often induce persistent cool and wet climate over global lands, altering and in some case mitigating regional climate warming impacts. The latest event lingered from mid-2010 to early 2012 and brought about intensive precipitation over many land regions of the world, particularly Australia. This resulted in a significant drop in global mean sea level despite the background upwards trend. This La Niña event is surprisingly predicted out to two years ahead in a few coupled models, even though the predictability of El Niño-Southern Oscillation during 2002–2014 has declined owing to weakened ocean-atmosphere interactions. However, the underlying mechanism for high predictability of this multi-year La Niña episode is still unclear. Experiments based on a climate model that demonstrates a successful two-year forecast of the La Niña support the hypothesis that warm sea surface temperature (SST) anomalies in the Atlantic and Indian Oceans act to intensify the easterly winds in the central equatorial Pacific and largely contribute to the occurrence and two-year predictability of the 2010–2012 La Niña. The results highlight the importance of increased Atlantic-Indian Ocean SSTs for the multi-year La Niña’s predictability under global warming.

## Introduction

The El Niño-Southern Oscillation (ENSO) represents the strongest interannual variability of tropical climate and often has large environmental and socio-economic effects worldwide. Prevailing theories have suggested that ENSO is governed by large-scale ocean-atmosphere dynamical interactions in the tropical Pacific, such as the Bjerknes feedback between sea surface temperature (SST), trade winds, and the ocean’s thermocline in the equatorial Pacific^1, 2^. For instance, an initial SST increase in the eastern Pacific tends to slacken the easterly trade winds and deepen the ocean’s thermocline, which in turn intensifies the initial increase in SST and thus leads to an El Niño event. This ocean-atmosphere interaction, however, does not lead to a perpetual El Niño condition. Instead, the slackened easterly winds during the growing El Niño act to decrease the equatorial Pacific basin-mean upper-ocean heat content, which eventually stops the growth of El Niño and may initiate the development of La Niña in following year^2, 3^. The interannual ENSO cycle, induced by this recharge-discharge mechanism^3^, not only strongly impacts regional climate but also strongly modulates the global mean surface temperature, exaggerating or mitigating the global warming forced by increased radiative forcing.

**Figure 1:**
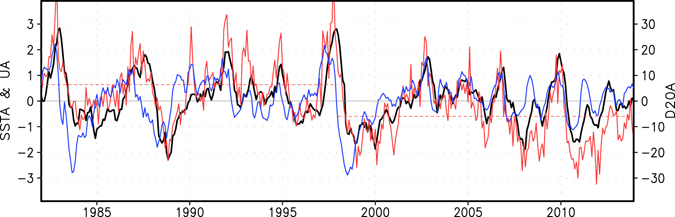
Observed ENSO-related SST (°C), zonal wind (m/s), and 20°C isotherm depth (D20, meter) anomalies during 1982–2013. The black line denotes the SST anomalies in Nino3.4 region (5°S–5°N, 170°W–120°W). The blue line indicates the warm water volume index^39^, represented by the D20 anomalies averaged in the equatorial Pacific basin (5°S–5°N, 150°E–90°W). The red sloid line displays the zonal wind anomalies in the central Pacific Ocean (5°S–5°N, 150°E–160°W). The dashed red lines denote the mean zonal wind anomaly of the period 1982–1997 and 1998–2013, respectively. This figure is created using Grid Analysis and Display System (GrADS) Version 2.0.2 (http://cola.gmu.edu/grads/).

**Figure 2:**
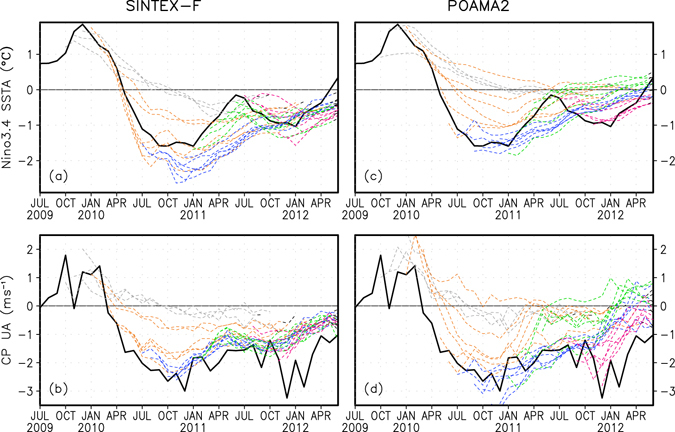
Two-year hindcasts of the La Niña in 2010–12 produced by the two global ocean-atmosphere coupled models. The black lines in (**a,c**) and (**b,d**) indicate the observed SST anomalies in Nino3.4 region and the zonal wind anomalies in the central Pacific Ocean, respectively. The dashed colour lines display plumes of the models’ two-year hindcasts of the SST and wind anomalies, which are initiated from every month during October 2009-December 2011 (see Methods). Results of the SINTEX-F and POAMA2 are based on 9 and 20 member ensemble mean hindcasts, respectively. The model hindcast anomalies are computed relative to the model climatology of 1983–2011 at each lead time. This figure is created using Grid Analysis and Display System (GrADS) Version 2.0.2 (http://cola.gmu.edu/grads/).

The ENSO cycle is not regular^4^. While El Niño’s onset often requires additional forcing such as intra-seasonal westerly wind bursts^5, 6^ and hence is less predictable, the transition from El Niño to La Niña is generally consistent with the recharge-discharge mechanism^3^ and displays high predictability^7^. In addition, La Niña usually peaks in the central Pacific and persists for a longer period than El Niño does^8, 9^. Strong westerly anomalies in the Pacific Ocean induced by the super El Niño event in 1982/83 and 1997/98 generated a strong discharge of heat away from the equator, and thus resulting in a cold basin-mean state in the equatorial Pacific; this provided a precursor for the occurrence of a multi-year lingering La Niña during late 1983-early 1986 and late 1998-early 2001, respectively (Fig. 1). These two La Niñas are predicted by a fully coupled global model at lead times of up to two years^7^. It has been recognized that La Niña episodes following weak-moderate El Niños with a weakened discharge process appear to be less predictable in general^7^. Surprisingly, the double-peaked La Niña during late 2010-early 2012 following a moderate (not strong) El Niño in 2009/10 is successfully predicted by the IOCAS intermediate coupled model^10, 11^ and two fully coupled models out to two years ahead (Fig. 2 and see Supplementary Fig. 1 for real time forecasts released online). This La Niña episode decreased the global surface temperature, brought about intensive precipitation over many regions and increased terrestrial water storage, particularly over Australia. This caused a 5 mm drop in global mean sea level, opposing to its long-term rise in response to global warming^12^.

## Results

Initiated from late 2009 to early 2010 (i.e., the peak of the El Niño), ensemble mean forecasts of two fully coupled models^7, 13^ that use different initialization approaches (see Methods) display a reasonably good skill in predicting the transition from El Niño to La Niña (Fig. 2a,c), albeit with a common bias of a delayed phase transition. When initiated from early-middle 2010 (i.e., the decay of the El Niño), the two models correctly predict a two-year lasting La Niña during late 2010-early 2012, despite that the second intensification in late 2011 is not well predicted. The La Niña onset is relatively better predicted in the SINTEX-F model but the intensity of the La Niña appears to be overestimated in some forecasts. Initiated from early 2011, the second but weak peak in Dec-Feb 2011/12 of this long-lasting La Niña is also predicted reasonably well in the SINTEX-F model. In addition, the strong rainfall anomalies during Sep-Nov 2010 and Dec-Feb 2010/11 for vast areas of Australia that caused severe floods and socio-economic losses were also predicted in real time forecasts (Supplementary Fig. 2). The high and long-lead predictability of the 2010–12 La Niña event is surprising, since many existing prediction systems have suggested a low predictability of ENSO (including La Niñas) during 2002–2014, compared to those in previous decades^14–16^. The decreased predictability during 2002–2014 may be dynamically linked with a weakened air-sea coupling in the equatorial eastern Pacific^17, 18^, so that the ocean’s subsurface signal does not provide a strong and effective precursor (Fig. 1). Another relevant factor is that ENSO intensity has weakened with a shortened periodicity and El Niño-related SST warm anomalies are more frequently shifted westward to the central Pacific^19–21^ during 2002–2014, probably partly related to a phase change of the Atlantic multidecadal oscillation^22^. These are distinctly different to the characteristics of canonical ENSO in previous decades.

Disentangling the mechanism underpinning the enhanced predictability of the 2010–12 La Niña is of importance in improving the understanding and multi-annual prediction of ENSO. Our analysis shows that, compared to the La Niña events in previous decades, the La Niña event in 2010–12 is accompanied with unusually strong easterly anomalies in the central Pacific Ocean, partly associated with an easterly mean-state shift in the late 1990’s (Fig. 1)^20, 21^. Note that this climate regime shift, which is statistically significant at the 1% level according to Student-t test, has been found to be linked with a decadal/multi-decadal Pacific variability^23^ and a rapid SST warming in the tropical Atlantic and Indian Oceans^21, 24–26^. The resultant inter-basin warming contrast between the Indian Ocean/Atlantic Ocean and the Pacific Ocean may have contributed to the intensified Walker circulation in the Pacific Ocean during the recent decades. The monthly mean easterly anomalies in the central Pacific Ocean persisted from mid-2010 to early-2012 and remained fairly strong even when the La Niña-related cold SST anomalies were weakened during April-October 2011. This suggests that parts of the strong easterly anomalies may not be generated by local ocean-atmosphere interactions in the Pacific Ocean. The long-lasting easterly anomalies in the central Pacific during 2010–2012, which favour the occurrence of the multi-year La Niña event^9^, are predicted out to two years ahead particularly in the SINTEX-F model (Fig. 2b,d), except that the intensity of the easterly anomalies is generally underestimated. The results imply that the exceptionally strong and highly persistent easterly anomalies in the central Pacific Ocean may have played an important role in the occurrence and two-year predictability of the long-lasting La Niña in 2010–12.

**Figure 3:**
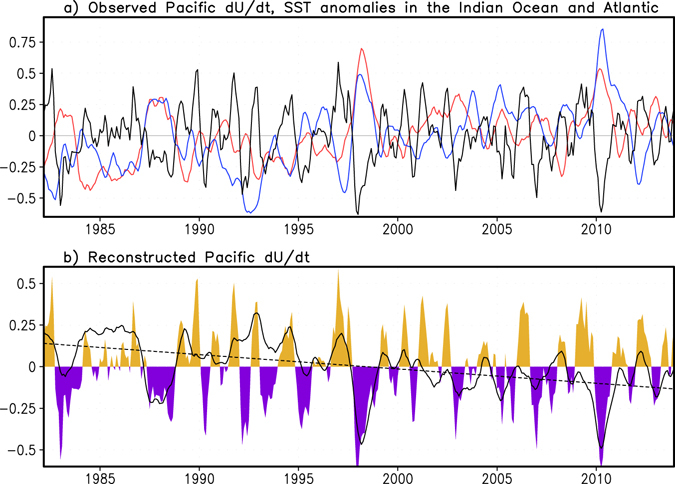
Observed and reconstructed zonal wind anomaly tendency in the central Pacific Ocean. (**a**) The observed zonal wind anomaly tendency in the central Pacific Ocean (dU/dt, black line), the SST anomalies in the tropical Indian Ocean (red line, 20°S–20°N, 40°E–120°E) and Atlantic Ocean (blue line, 20°S–20°N, 70°W–15°E). Results are based on 5-month running mean anomalies. (**b**) The observed dU/dt (colour filled line) and reconstructed dU/dt (black solid line, with the linear trend being included) in the central Pacific Ocean based on the SST anomalies in the Indian Ocean and Atlantic Ocean (see Methods). The black dashed line denotes the linear trend component of the reconstructed dU/dt that displays an easterly tendency trend forced by the rapid SST warming in the Indian Ocean and Atlantic Ocean. This figure is created using Grid Analysis and Display System (GrADS) Version 2.0.2 (http://cola.gmu.edu/grads/).

We find that the easterly wind tendency in the central Pacific Ocean is significantly correlated with SST anomalies in both the tropical Indian Ocean and Atlantic Ocean (Fig. 3a), with the maximum correlations being −0.47 and −0.40 at zero month lead/lag, respectively (both are statistically significant at 1% level according to Student-t test). Based on a multiple regression model (see Methods), correlation of the central Pacific wind tendency predicted by the detrended Atlantic and Indian Ocean SST anomalies and the original wind tendency reaches 0.59. That means, nearly 36% of the variance of the observed central Pacific wind tendency can be statistically explained by the detrended SST anomalies in the other two oceans. According to this multiple regression model, the rapid SST warming in the Indian Ocean and Atlantic Ocean in recent decades has caused an easterly tendency trend in the central Pacific Ocean, consistent with previous studies^21, 24–26^. In addition, the strong and lingering easterly tendency in 1987–88, 1997–98, and 2010 are also reproduced (Fig. 3b). During the multi-year La Niña episode in 2010–12, the warm SST anomaly in the Atlantic Ocean persisted from 2009 to late 2011 and reached unprecedented warmth of about 0.8 °C during March-June 2010 (Fig. 3a and Supplementary Fig. S3). Similarly, the SST in the Indian Ocean also persisted above normal during 2009–2012, except a short period of January-May 2011 during which the Indian Ocean SST anomaly became negative in association with the strong La Niña’s influence^27^. The SST anomalies in both the Indian Ocean and Atlantic Ocean during 2010–2011 are predicted by the two global climate models (Supplementary Fig. S3), except that the warm SST anomalies in the Atlantic Ocean during April-July 2011 are underestimated.

**Figure 4:**
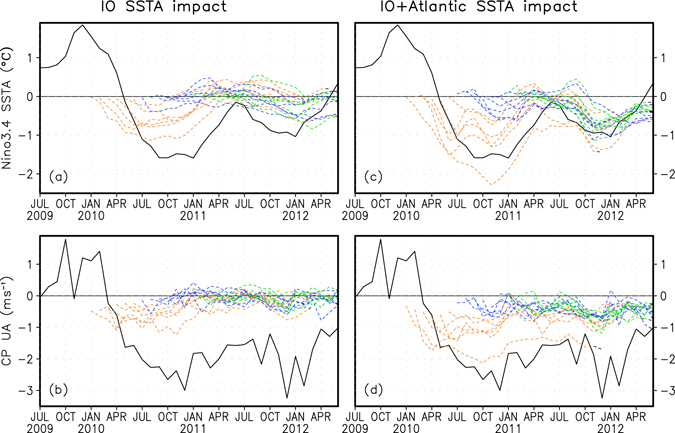
Impacts of the SST anomalies in the Indian Ocean and Atlantic Ocean on the predictability of the La Niña in 2010–12. (**a**,**b**) As in Fig. 2a and b, but for the differences in the first group of two sensitivity experiments in which observed climatological SST and observed monthly SST values are prescribed respectively in the Indian Ocean during the model’s two-year predictions (Methods). (**c,d**) As in (**a,b**), but for results based on the second group of two sensitivity experiments in which observed climatological SST and observed monthly SST values are prescribed respectively in both the Indian Ocean and the Atlantic during the model’s two-year predictions. Results are based on 9-member ensemble mean predictions. This figure is created using Grid Analysis and Display System (GrADS) Version 2.0.2 (http://cola.gmu.edu/grads/).

To clarify the importance of the warm SST anomalies in the Atlantic and Indian Oceans for the multi-year La Niña episode in 2010–12, we conduct two groups of prediction experiments with the SINTEX-F model (see Methods). Each group consists of two sensitivity experiments. In the first group, we specify observed climatological SST in one experiment and observed monthly SST values in the other experiment in the Indian Ocean and then re-run the two-year predictions initiated from each month during Jan 2010–Dec 2011. The differences of the two sensitivity experiments represent the influences of the Indian Ocean SST anomalies on the La Niña prediction. In the second group of the model sensitivity experiments, we specify climatological SST in one experiment and observed SST values in the other experiment in both the Indian Ocean and Atlantic Ocean, aiming to explore the combined influence of the two oceans. The results suggest that the SST anomalies in the two oceans jointly play an important role and account for considerable parts of the observed double-peaked La Niña SST anomalies and the persistent easterly anomalies in the central Pacific Ocean during 2010–12 (Fig. 4). Their combined contributions, however, generally do not fully explain the observed intensities related to this La Niña, and are also smaller than those of the model hindcasts (recall Fig. 2). This suggests that other factors such as local Pacific ocean-atmosphere interactions and/or persistent cooling subsurface effects associated with off-equatorial thermal advection^28^ may also play an important role. Note that many models tend to underestimate the inter-basin influence of the Indian Ocean and Atlantic on the Pacific^29^; this bias may also affect the estimation of the contributions of the other two basins to the La Niña in 2010–12.

Similar to the multi-year La Niña episode in 2010–12, the double-peaked La Niña in 2007–09, following a weak-to-moderate El Niño in 2006/07, is also linked with strong and persistent easterlies in the central Pacific Ocean (Fig. 1). The easterlies are not as strong as those in 2010–12, possibly in association with weak warm SST anomalies in the Indian Ocean and Atlantic Ocean (Fig. 3). The two models display a low two-year skill in predicting both this La Niña event (Supplementary Fig. 4) and the weak SST anomalies in the Indian and Atlantic Oceans (Supplementary Fig. 5). Correspondingly, results based on another two groups of two model sensitivity experiments suggest small contributions of the SST anomalies in the Indian and Atlantic Oceans to the La Niña development in 2007–09 (Supplementary Fig. 6).

## Discussions

Previous studies have suggested that the SST anomalies in the Indian and Atlantic Oceans may contribute to the phase transition and predictability of ENSO at multi-month or multi-season time scales^30–35^. Both the Indian Ocean and Atlantic SST anomalies can affect the Pacific via modifying the east-west Walker circulations that connect the three tropical oceans^30–36^. Besides, it has also been suggested that the Atlantic SST anomalies can influence the Indian Ocean that in turn affects the Pacific^26^. Our results further demonstrate that the multi-year persisting warm SST anomalies in the Indian Ocean and Atlantic, in response to a preceding El Niño as well as decadal/multi-decadal variations and/or global warming^27, 37^, contribute to the occurrence and two-year predictability of double-peaked La Niñas, particularly in the 2010–12 case. This multi-year La Niña episode happened despite that the ENSO-related dynamical coupling has generally weakened during 2002–2014^17, 18^. Improved understanding and simulation of the warm SST anomalies in the Indian and Atlantic Oceans may improve multi-annual prediction and projection of long-lasting La Niñas in the future.

It is worth noting that strong warm SST anomalies also occurred in the Indian Ocean and Atlantic during 1998 (recall Fig. 3a), in association with the remote influence of the supper 1997/98 El Niño event^27^. Consistent with previous studies^30–34^, our results suggest that the strong warm SST anomalies in the two oceans may also contribute to the strong easterly tendency in the central Pacific in 1998 (recall Fig. 3b). It has been found that the warm SST anomalies in the Indian Ocean and Atlantic during 1998 may favour the rapid phase transition from El Niño to La Niña^30–34^. However, even if the Indian Ocean and Atlantic SST anomalies were suppressed in model prediction experiments, the multi-year La Niña event in late 1998-early 2001 would still occur^31, 38^. Besides, it has been found that impacts of the Indian Ocean SST anomaly on ENSO prediction skill during 1982–2006 are modest in general^15^. These suggest that the occurrence and multi-year predictability of the 1998–2001 La Niña event may be mostly controlled by the local recharge-discharge mechanism in the Pacific.

Three distinctive differences are visible between the two multi-year La Niña events in 1998–2001 and 2010–12. The first difference is that the 1998–2001 La Niña has a strong cold ocean subsurface precursor, in association with the strong discharge process of the super 1997/98 El Niño (recall Fig. 1). In contrast, there is no clear ocean subsurface precursor for the 2010–12 La Niña event. The second difference is that the warm SST anomalies in the two oceans (particularly in the Atlantic) during the 2010–12 La Niña event are stronger and persist longer than those during the 1998–2001 La Niña event (i.e., Fig. 3a). The third difference is that the 2010–12 La Niña event is linked with unusually strong easterly anomalies in the central Pacific Ocean, compared to the 1998–2001 La Niña event (i.e., Fig. 1). Distinct features can also be seen in the 1983–86 La Niña case. Future studies are warranted to explore the relative roles of the inter-basin influences and the Pacific local recharge-discharge mechanism in the occurrence and predictability of the multi-year La Niña events in previous decades.

## Methods

### Observations and multiple regression method

We use the Reynolds sea surface temperature (SST) data that combines both *in situ* (ship and buoy) and satellite observations from 1982 to 2013 (ref. 40). Observational surface winds are obtained from the National Centers for Environmental Prediction (NCEP) reanalysis^41^, precipitation from Global Precipitation Climatology Project (GPCP) Version 2.2 (ref. 42), and the ocean reanalysis from the NCEP global data assimilation system^43^. Observed anomalies are calculated relative to the monthly climatology of 1983–2011.

The zonal wind tendency (dU/dt) in the central Pacific Ocean (5°S–5°N, 150°E–160°W) shows significant correlation with SST anomalies in the tropical Indian Ocean (IO, 20°S–20°N, 40°E–120°E) and Atlantic Ocean (20°S–20°N, 70°W–15°E). We build a simple multiple-regression model: dU/dt = a * IO SST anomaly + b * Atlantic SST anomaly + c. Since the dU/dt has automatically removed the linear trend component, detrended SST anomalies in the two oceans are used to calculate the multiple regressions. Based on the 5-month running mean observations during 1982–2013, we get: a = −0.472 and b = −0.287 (unit: m s^−2^ K^−1^), and the constant c = 0.002. The correlation between the original dU/dt and the reconstructed one is 0.59, statistically significant at the 1% level according to Student-t test.

### Model hindcasts

Model ensemble hindcasts initiated from every month during 1982–2011are performed using two global ocean-atmosphere coupled climate models: the Japanese SINTEX-F^7^ and Australian POAMA2^13^. The SINTEX-F provides 9-member ensemble predictions that are generated by three different initial conditions for each of three model versions with modified coupling physics^7^. Only the observed SST values are assimilated in the coupled model to generate realistic and well-balanced initial conditions required for the hindcasts. This model has shown useful skill in predicting ENSO during 1982–2005 at lead times of up to two years^7^. In particular, the two multi-year La Niña events during late 1983 to early 1986 and during late 1998 to early 2001, following the two super El Niños in 1982/83 and 1997/98, are predicted at lead times of up to two years^7^. Their long-lead predictability is in accordance with the ENSO recharge-discharge theory^2, 3^. The latest long-lasting La Niña in 2010–12 and its induced floods in Australia are also forecasted in real time out to two years ahead (Supplementary Figs 1 and 2, also see www.jamstec.go.jp/frcgc/research/d1/iod/e/seasonal/outlook.html).

The POAMA model provides 20-member ensemble predictions that are generated by 10 different initial conditions for each of two model versions with modified physics^13^. Both the atmospheric reanalysis and ocean observations are assimilated in the model to generate the initial conditions. The model has shown useful skill in predicting ENSO at lead times of 9 months^44^. Because of the limited computation power, only the central member of each of the two model versions is used to conduct two-year hindcasts, which are initiated from every month during 1982–2011, to produce the model’s forecast climatology up to two years lead. Initiated from every month during Oct 2009-Dec 2011 and Oct 2006-Dec 2008, two-year forecasts with 20-member ensembles are performed to examine the predictability of the two multi-year La Niña events in 2010–12 and 2007–09. The forecast anomalies of each member of the two model versions are calculated by the differences between each member and the forecast climatology of the central member of the same model version. The two-year prediction plumes are produced by the ensemble mean of all the members. Note that all forecast anomalies of the two models are computed relative to their climatology of 1983–2011 at each lead time.

### Model sensitivity experiments

To explore possible impacts of the SST anomalies in the tropical Indian Ocean and Atlantic Ocean on the two-year predictability of the La Niña in 2010–12, we conduct two groups of prediction experiments using SINTEX-F. Each group consists of two sensitivity experiments. In the first group of the model experiments, we specify observed climatological SST of 1983–2011 in one experiment and observed monthly SST values in the other experiment in the tropical Indian Ocean (20°S–20°N, 40°E–120°E)^31^ during the two-year sensitivity predictions with 9-member ensembles. The two-year sensitivity predictions are initiated from every month during Jan 2010-Dec 2011 using the same initial conditions as those used in the 9-member ensemble two-year hindcast experiments. Thus, the differences in the two sensitivity prediction experiments represent the impacts of the Indian Ocean SST anomalies on the predictability of the La Niña in 2010–12.

In the second group of the model experiments, we specify observed climatological SST in one experiment and observed monthly SST values in the other experiment in both the tropical Indian Ocean and Atlantic Ocean (20°S–20°N, 70°W–15°E) in the model’s two-year sensitivity predictions with 9-member ensembles. The differences in these two sensitivity predictions represent the combined impacts of the SST anomalies in both the Indian Ocean and Atlantic Ocean on the predictability of the La Niña in 2010–12.

In addition, similar to the above two groups of two experiments during Jan 2010-Dec 2011, we conduct another two groups of two sensitivity prediction experiments during Jan 2007-Dec 2008 with 9 members. This is to examine the impacts of SST anomalies in the Indian Ocean and the combined impacts of SST anomalies in both the Indian Ocean and Atlantic Ocean on the predictability of the La Niña in 2007–09. Results shown are based on 9-member ensemble mean of the model’s sensitivity predictions (Fig. 4 and Supplementary Fig. 6).

## Electronic supplementary material

supplementary information
