# Supplementary material for: Inter-basin sources for two-year predictability of the multi-year La Niña event in 2010–2012
Source: Sci Rep. 2017 May 23;7:2276. doi: 10.1038/s41598-017-01479-9 (PMC5442129; doi:10.1038/s41598-017-01479-9)
Supplement: Supplementary file 1 — supplementary information [file 41598_2017_1479_MOESM1_ESM.pdf]

Supplementary information:

Inter-basin sources for two-year predictability of the multi-year La Niña event in 2010-2012

Jing-Jia Luo[1*], Guoqiang Liu[1], Harry Hendon[1], Oscar Alves[1] & Toshio Yamagata[2]

[1]Bureau of Meteorology, Melbourne, Australia

[2]Application Lab, JAMSTEC, Yokohama, Japan

[*]Corresponding author: Dr. Jing-Jia Luo

Bureau of Meteorology

Level 9, 700 Collins Street, Docklands, Melbourne, VIC 3008, Australia

Email: j.luo@bom.gov.au

Tel: +61-3-9669-4235

Fax: +61-3-9669-4660

This supplementary information file includes:

Supplementary Figures 1-6

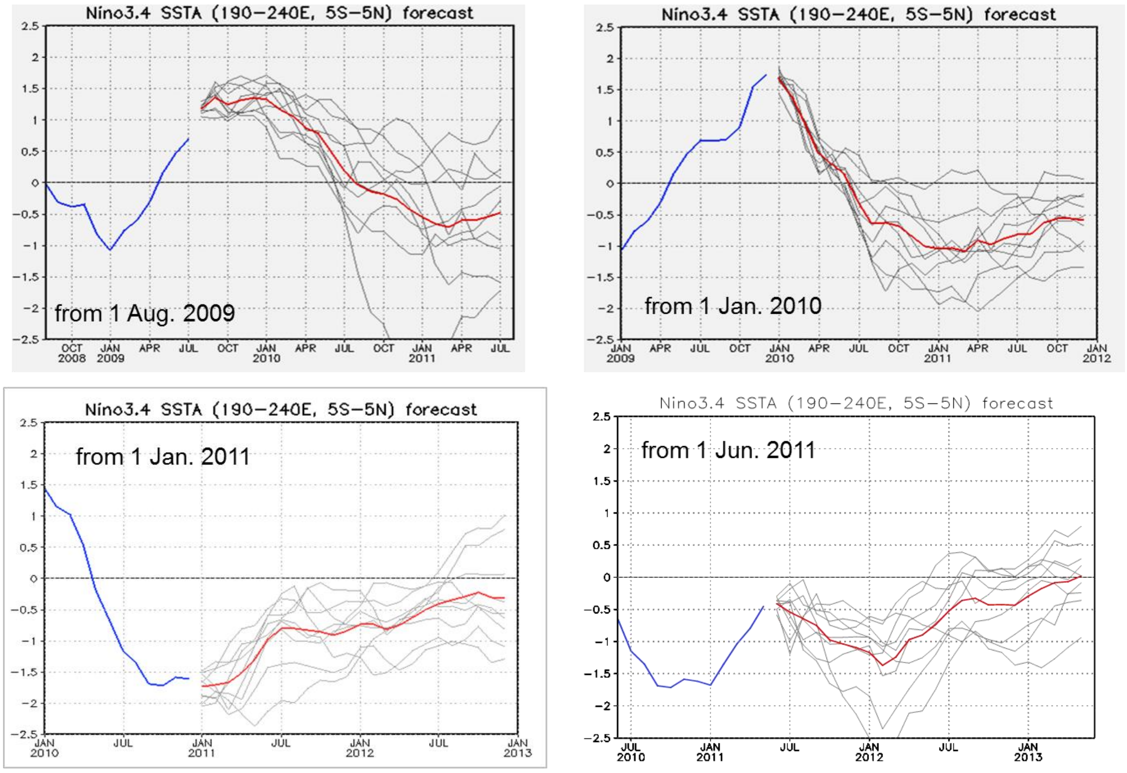

Supplementary Figure 1: **Real time two-year forecasts of the La Niña in 2010-12 based on the SINTEX-F model.** Blue lines denote the observed SST anomalies available up to the start dates of each month's real time forecasts. Grey (red) lines indicate the individual member (9-member ensemble mean) forecasts out to two years ahead. The real time forecasts initiated from every month are available at www.jamstec.go.jp/frsgc/research/d1/iod/e/seasonal/outlook.html. This figure is created using Grid Analysis and Display System (GrADS) Version 2.0.2 (http://cola.gmu.edu/grads/).

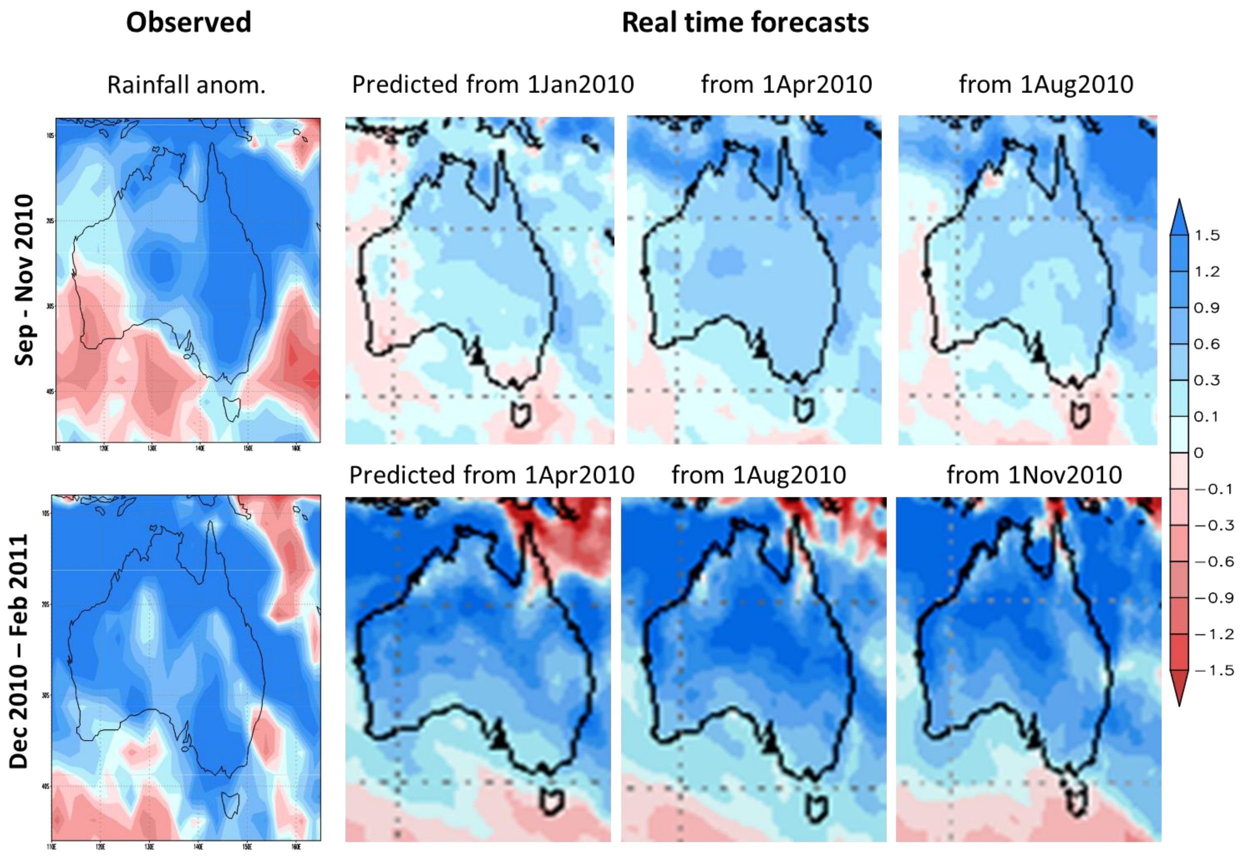

Supplementary Figure 2: **Real time forecasts of precipitation anomalies (mm/day) over Australia during Sep-Nov 2010 and Dec-Feb 2010/11.** The observed anomalies are produced based on GPCP version 2.2 products and the forecasts are made by the SINTEX-F model. The real time forecasts initiated from every month are available at www.jamstec.go.jp/frsgc/research/d1/iod/e/seasonal/outlook.html. This figure is created using Grid Analysis and Display System (GrADS) Version 2.0.2 (http://cola.gmu.edu/grads/).

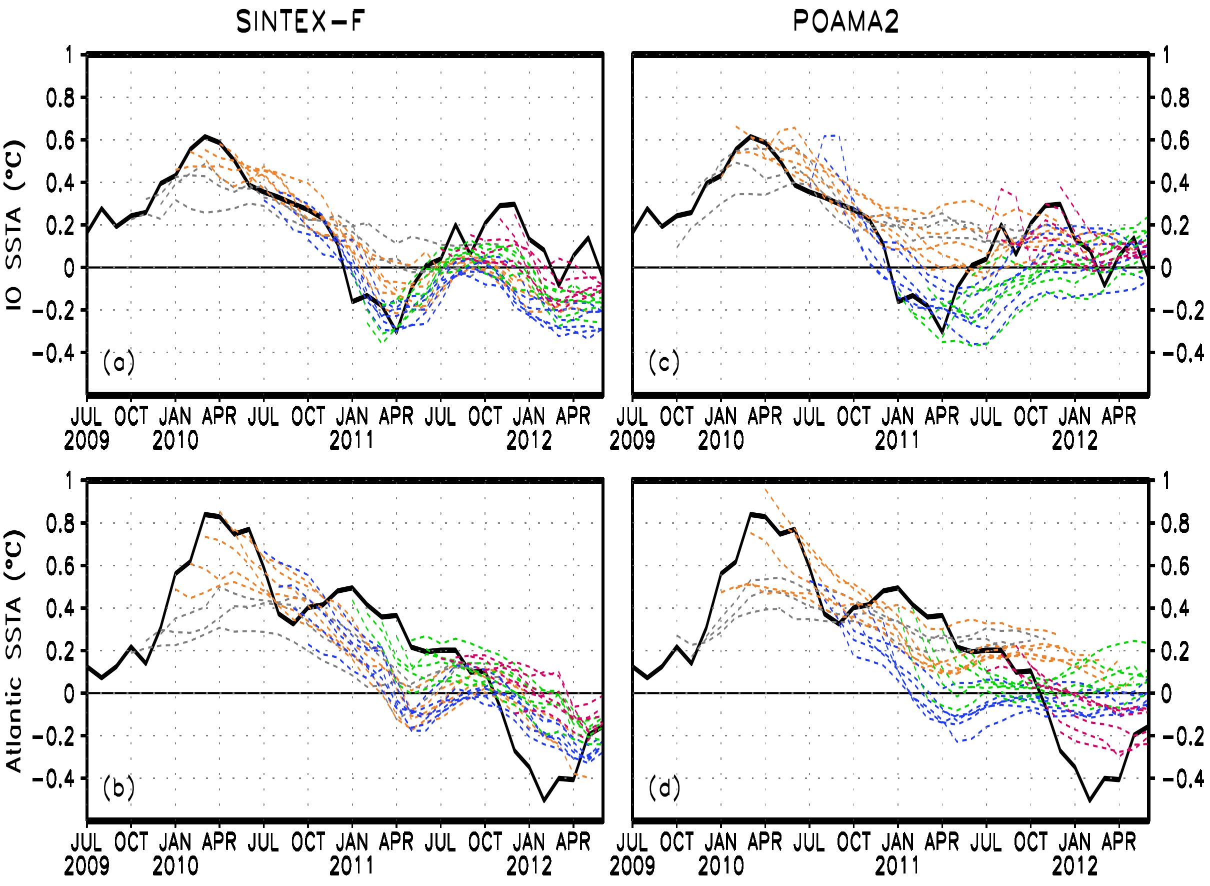

Supplementary Figure 3: **Two-year hindcasts of the SST anomalies in the tropical Indian Ocean and Atlantic Ocean in 2010-12.** The black lines in (**a,c**) and (**b,d**) indicate the observed SST anomalies in the tropical Indian Ocean (20°S-20°N, 40°E-120°E) and Atlantic Ocean (20°S-20°N, 70°W-15°E), respectively. The dashed colour lines display plumes of the models' two-year hindcasts, which are initiated from every month during Oct 2009-Dec 2011. Results of the SINTEX-F and POAMA2 model are based on 9 and 20 member ensemble mean hindcasts, respectively. This figure is created using Grid Analysis and Display System (GrADS) Version 2.0.2 (http://cola.gmu.edu/grads/).

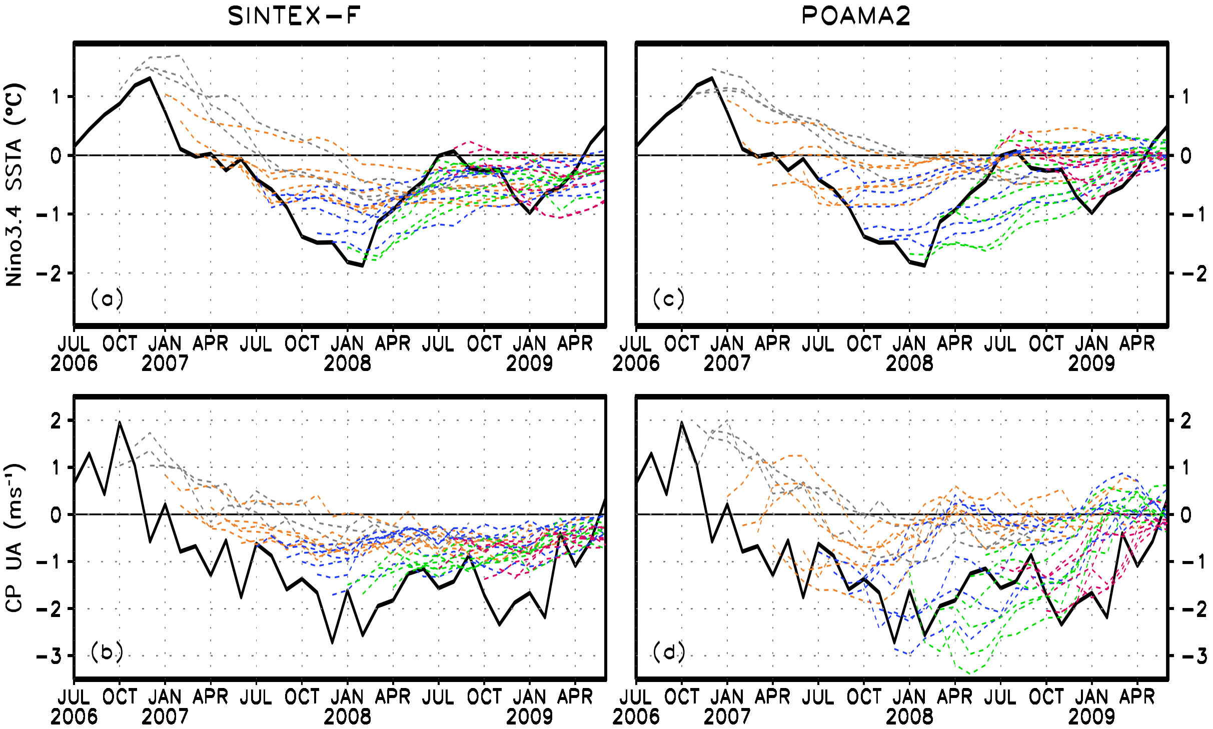

Supplementary Figure 4: **Two-year hindcasts of the La Niña in 2007-09 produced by the two global ocean-atmosphere coupled models.** As in Fig. 2, but for the results of the La Niña event in 2007-09. This figure is created using Grid Analysis and Display System (GrADS) Version 2.0.2 (http://cola.gmu.edu/grads/).

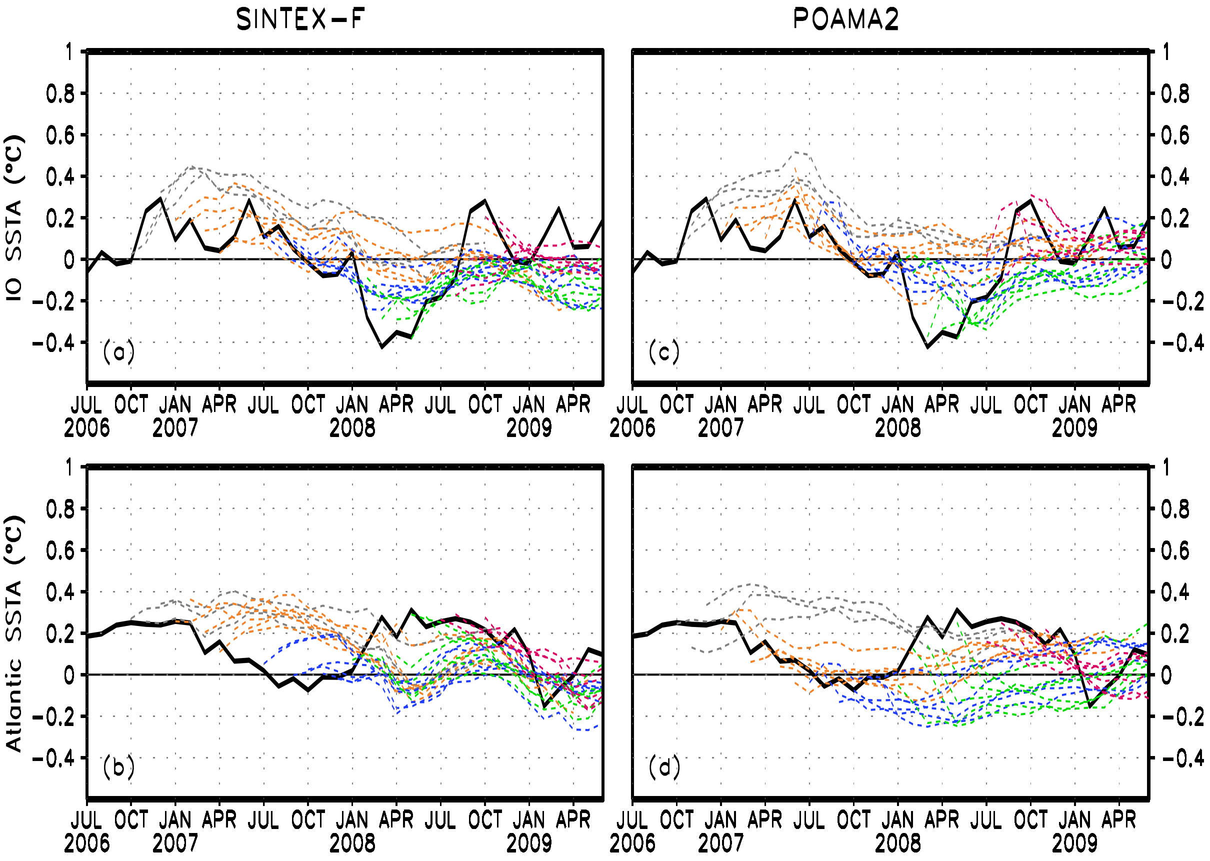

Supplementary Figure 5: **Two-year hindcasts of the SST anomalies in the tropical Indian Ocean and Atlantic Ocean in 2007-09.** As in Supplementary Fig. 3, but for the results in 2007-09. This figure is created using Grid Analysis and Display System (GrADS) Version 2.0.2 (http://cola.gmu.edu/grads/).

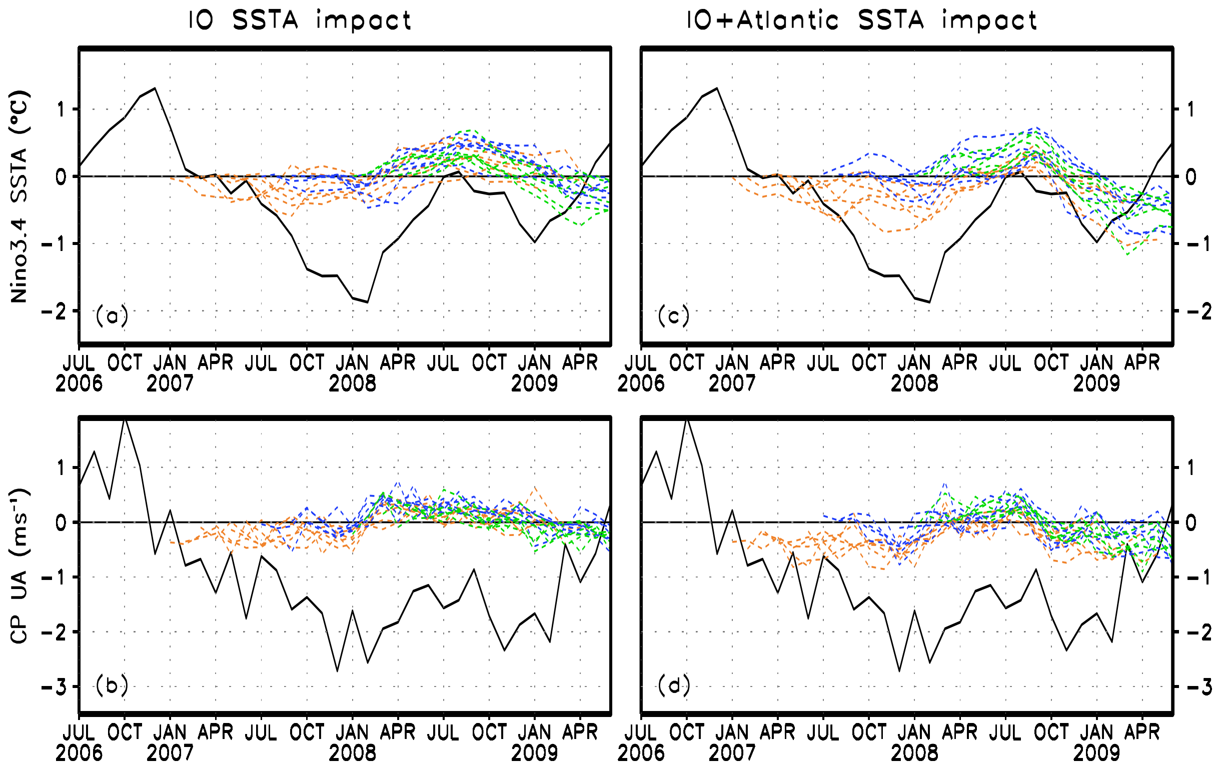

Supplementary Figure 6: **Impacts of the SST anomalies in the tropical Indian Ocean and Atlantic Ocean on the predictability of the La Niña in 2007-09.** As in Fig. 4, but for the results of the La Niña in 2007-09. This figure is created using Grid Analysis and Display System (GrADS) Version 2.0.2 (http://cola.gmu.edu/grads/).
